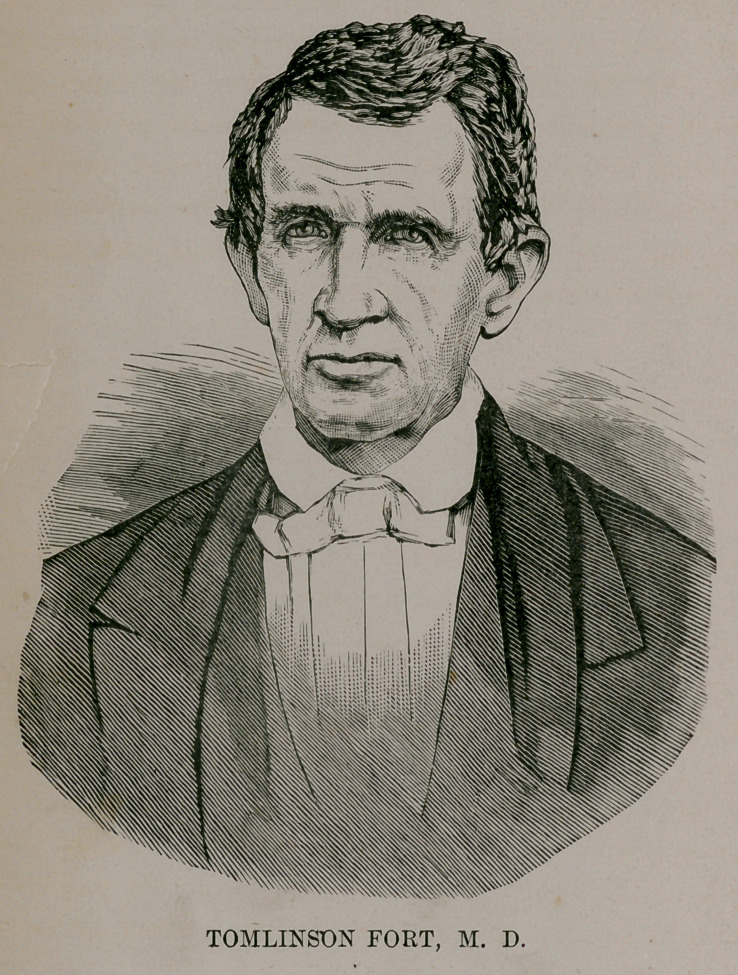# Our Portrait Gallery

**Published:** 1885-06

**Authors:** Junius Hillyer

**Affiliations:** Decatur


### OUR PORTRAIT GALLERY.

### TOMLINSON FORT, M. D.

Our picture gallery is ornamented this month with the likeness of one of the most distinguished and successful physicians who has ever lived in the State of Georgia.

Dr. Tomlinson Fort was of English ancestry. His father, Hon. Arthur Fort, came to Georgia when a young man, before the revolutionary war, and was an active participant in the stirring scenes of that eventful period. As a member of the Committee of Safety; as a soldier in the field against British, Tories and Indians; as a member of the Legislature, he gave to the patriots' cause and to his country the benefit of his clear intellect, his true heart and his strong arm.

He married a Mrs. Whitehead (*nee* Tomlinson), of Burke county, and reared a numerous family of children, the fourth of whom, named after his mother's family, Tomlinson, is the subject of this sketch.

Dr. Fort was born in 1787, precisely coeval with the constitution of the United States. After the then usual period of apprentisage or private pupilage, he repaired to Philadelphia and prepared himself for graduation in the University of Pennsylvania, under the tuition of Rush, Physic and their confreres, who then illustrated that renowned institution.

Returning to Georgia, he settled in Milledgeville, the capital of the State, where he spent his entire professional life. His success came early and knew no diminution. His dignified manners and his absolute integrity inspired confidence and respect, and a peculiar magnetism drew to him the strong, personal attachment of all with whom he came in contact. His reputation was not long confined to the town or county of his residence, but extended widely over the State, and few, if any, of the physicians of the State have ever gained so large a clientage or such honorable distinction. Dr. Fort brought to bear in his practice a well-balanced mind, a large stock of common sense, keenness of observation and a power of analysis, which enabled him to judge truly of popular superstitions, reckless assertions of authors, and to reject, when he deemed them erroneous, the dogmas, even of his favorite teacher, Dr. Rush. To the revolution in the practice of physic which occurred during his life, which delivered us from the ravages of mercury and salivation, from the dangerous, indiscriminate use of the lancet, and from the horrible torture of days of fever without the solace of a single drop of cold water, Dr. Fort contributed his full share.

He was not a voluminous contributor to the periodical, professional literature of the period, but late in life he published a volume of some seven hundred pages, which he modestly called a "Dissertation on the Practice of Medicine." This book he dedicated to the physicians of the State of Georgia, as a grateful acknowledgment of the kindness, respect and confidence which he had experienced at the hands of every one of them with whom he had the honor of becoming acquainted. This work, he says, "is, in its nature, ephemeral." Its author does not claim for it a place among the standard works of the day, but some of the more important diseases are treated at considerable length and with great ability. It exhibits throughout, the care, candor, acumen, originality and courage of conviction of its author. No physician can read it without instruction, or finish its perusal without the highest respect for his moral as well as professional characteristics. As a book for the guidance of families, where good medical advice is not attainable, it is held in high esteem.

Dr. Fort's popularity and influence in the city of his residence were overwhelming. The first case of small-pox that ever occurred there was under his care. He gave to it the conscientious attention he deemed requisite, but the alarm in the community was so great that neither nurse nor shelter could be procured, and the doctor furnished one and became the other. The alarmed citizens, in a town meeting, resolved to compel him, by violence, if necessary, to desist from his attentions. He quietly placed a loaded gun at his door and notified them that he would permit no one to interrupt the discharge of his professional duties. When the danger was passed, the fickle mob again met and passed a resolution of thanks, complimenting him on his courage and fidelity.

The sufferer was a son of Governor Clarke, whose family presented the doctor with a magnificent service of silver-plate as a token of gratitude.

Laborious in his profession, as he was for many years, he was not unmindful of any of the duties of citizenship. In the war of 1812, he raised and commanded a company, and received in battle a wound in the knee, which gave him great suffering during the remainder of his life.

He represented Baldwin county eight years in the halls of legislation, and the State two years in the Congress of the United States.

A sketch of his political character and standing, kindly furnished by his distinguished and venerable friend, the Hon. Junius Hillyer, is subjoined.

Of the life in Washington his then young wife writes as follows: "Two years after our marriage my husband was sent to Congress. I went with him, taking our only child, Julia, going in our own carriage.

Those two years in Washington were bright ones and are vividly remembered. I met many agreeable and cultivated people, boarded with the Bigelows of Boston, and Mr. and Mrs. Edward Everett. He was a charming man, and she a very nice, though not remarkable person. I met, of course, the best people in the land; dined more than once at the President's (Adams), and saw a great deal of society. I met Henry Clay, with his bright blue eye and eloquent tongue, and Mrs. Clay, a good but plain woman, whom he had married in obscurity and afterwards outgrown. Also Webster, who, with his great head and solemn ways, was not a favorite with women. But the greatest of all to me was my own and my husband's friend, John C. Calhoun. Such a brilliant eye and such fascinating manners I have never seen since.

I saw the inauguration of President Jackson. His progress up the avenue on horseback was simple, yet dignified."

At the close of his term in Congress, Dr. Fort retired from active political life; the wants of a growing family and the expenses of a profuse and generous hospitality demanded the resources of his large professional income, and he sacrificed a most brilliant public career upon the altar of domestic and social obligation.

Dr. Fort, in 1824, married Miss Martha Fannin, one of the most admired and accomplished belles of the elegant society which at that time existed in Middle Georgia. The Fannin family, already distinguished in Ireland before their emigration to the colonies, has representatives in nearly every State from Canada to Texas, the men always noted for patriotism and personal valor, the women for intelligence and personal beauty. The Georgia Fannins showed the ancestral traits, and none more notably than the fair representative who, at twenty years old, bound in indissoluble chains the grave, sedate, quaker-like bachelor of thirty-seven. As the readers of the JOURNAL are not ladies, it is useless to describe the quiet wedding or the bridal dress. The characteristic fact, however, is noted that the busy doctor forgot to order his swallow-tail coat of blue broadcloth, with large brass buttons, until almost too late to don it before the ceremony. The marriage was a happy one. Their home was established in Milledgeville, where they lived until the date of his death, 17th May, 1859.

During all that period he was at the summit of professional reputation, of social standing and political influence.

Dr. Fort left three sons and several daughters. The eldest son, Dr. George Fort, died shortly after his father. Col. Tomlinson Fort, a lawyer, and lately mayor of that city, lives in Chattanooga, unmarried. Col. John Fort, married to the accomplished sister ot the Hon. W. D. Ellis, of this city, lives near Macon and is engaged in agricultural pursuits. Two of the daughters died and one is unmarried. The youngest is the wife of Julius Brown, Esq., a most prominent lawyer, and an influential and wealthy citizen of the city of Atlanta.

### SKETCH OF DR. FORT BY JUDGE JUNIUS HILLYER.

*Dr. Miller:*

DEAR SIR—My knowledge of Dr. Tomlinson Fort commenced in 1828. I saw him in Milledgeville during the session of the Legislature in the fall of that year. In person he was tall, straight, symmetrical, and a form indicating endurance, health and a sound constitution. He had a sedate but cheerful, friendly expression that inspired his associates with respect and kind feeling towards him. And I do not believe that his feelings were ever wounded by his most bitter political opponents or by any of his personal associates. When I first knew him he was in the prime of manhood, perhaps between thirty-five and forty years of age. He was an active, working member of the old Clarke party, and was personally known by every prominent man in the State, for the men of both parties sought and valued his acquaintance. Dr. Fort was not numbered among the great orators of his day. I have often heard him speak in public. He rarely spoke over half an hour, and always kept close to the questions under consideration, and without any flourishes of rhetoric or effort at the beautiful, he gave his views in a plain, straightforward, earnest manner, which commanded the attention of his hearers, while everything he said was understood clearly, and it was no labor to listen to him and follow his line of thought. Such a speaker must necessarily command attention and wield an influence. Dr. Fort, as a party man, was a strong, important leader. He held the most extreme partisan views; he held, and always openly avowed, the good old Jackson democratic doctrine, that "to the victors belong the spoils." His party motto was, "Turn them out; put the government in the hands of the Democrats." As a partisan he was pre-eminently a Bourbon Democrat. He never learned any new principles and he never changed his old ones. He was a man of the people; he lived with the people; he guided their political ideas and moulded their judgments. In his party he preferred a position in the ranks of a private. He rarely sought office. I am sure he could have attained any office in the gift of the people if he had desired it. Here we have a man who began life in the midst of the angry strife of the Federal and Republican parties, participated actively in all the stirring scenes of the last war with England. The strife over the United States bank; the inauguration of the tariff policy; the bitter personal strife between the Clarke and Crawford parties and the Clarke and Troup parties; our controversies about the Indians and Indian lands; our angry strife about nullification and the Union; General Jackson's war on the United States bank; the sub-treasury; the war with Mexico and the acquisition of Texas; the slavery question and the compromise of 1850, stirring and moving the people through all these long years down to the time of his death, a period of half a century—all these scenes he witnessed. He mingled with the actors. He participated in the discussion of all these momentous questions with much crimination and recrimination, with many a duel and many a fight, and wide-spread hatred and life-long animosity, yet from it all he came forth in his old age out of this fiery ordeal without the smell of fire on his garments—universally beloved by all men of all parties. The reason is plain—he was wise, he was good, he was just, and he was polite.

Twelve years we were together on the Board of Trustees of the State University, and every year, for nearly thirty years, I saw him in Milledgeville, and often in other places, so I can say I knew him well. And I know his character, what his acquaintances say of him—of his private life. All can be said in one short line: *He stood through his long life above reproach.*

Through all the length and breadth of the State Dr. Fort was, in the judgment of all who knew him, in the first rank of his profession. More than one generation must pass away before, in Baldwin county, his skill, his patience and his kindness to the sick and to the poor will be forgotten.

Respectfully, JUNIUS HILLYER.

DECATUR, May 9th, 1885.

## Figures and Tables

**Figure f1:**